# Osteoarthritis in People with Multiple Sclerosis: A Systematic Review and Meta-Analysis

**DOI:** 10.3390/jcm13175015

**Published:** 2024-08-24

**Authors:** Vasileios Giannopapas, Vassiliki Smyrni, Dimitrios K. Kitsos, Sophia Stasi, Athanasios K. Chasiotis, Christos Moschovos, Georgia Papagiannopoulou, Konstantina Stavrogianni, Maria Kosmidou, Daphne Bakalidou, John S. Tzartos, Georgios Tsivgoulis, Sotirios Giannopoulos

**Affiliations:** 1 Second Department of Neurology, National and Kapodistrian University of Athens, 15772 Athens, Greece; bgiannopapas@gmail.com (V.G.); b.smyrni@hotmail.com (V.S.); dkitsos@icloud.com (D.K.K.); thanosch1@gmail.com (A.K.C.); moship@windowslive.com (C.M.); georgiapap22@hotmail.com (G.P.); stavrogianni.k@gmail.com (K.S.); dafbak@otenet.gr (D.B.); jtzartos@gmail.com (J.S.T.); tsivgoulisgiorg@yahoo.gr (G.T.); 2 Department of Physical Therapy, University of West Attica, 12243 Egaleo, Greece; 3 Department of Physical Therapy, University of Peloponnese, 23100 Sparti, Greece; soniastasi1@gmail.com; 4 Department of Physiology, Faculty of Medicine, University of Ioannina, 45110 Ioannina, Greece; 5 Department of Internal Medicine, Faculty of Medicine, University of Ioannina, 45110 Ioannina, Greece; mskosmidou@gmail.com

**Keywords:** multiple sclerosis, arthritis, osteoarthritis, autoimmune diseases

## Abstract

**Background:** Arthritis, particularly osteoarthritis (OA), is a common synovial condition observed in individuals with multiple sclerosis (MS). Despite its high prevalence and significant impact on the quality of life of MS individuals, there is a gap in the current literature regarding the prevalence of OA in this population and its relation to MS pathology. This systematic review and meta-analysis aimed to estimate the prevalence of OA in the MS population and explore potential associations with demographic and MS-specific characteristics. **Methods**: Adhering to PRISMA guidelines, a systematic search of the MEDLINE PubMed, Scopus and Google Scholar databases was conducted. **Results**: Fifteen studies were included in the systematic review and meta-analysis. The aggregated prevalence of OA in the MS population was 27% (95% CI: 15–40%), with substantial heterogeneity (I^2^ = 99.9%). Sensitivity analysis, excluding one study, showed a prevalence of 21% (95% CI: 16–28%). The risk ratio of OA in MS versus controls was 1.07 (95% CI: 0.84–1.37), indicating no significant difference. Meta-regression revealed no associations between OA prevalence and age or disease duration in MS patients. **Conclusions**: This study reports a 21–27% prevalence of OA in people with MS. Understanding the implications of OA in pain and mobility domains, as well as the challenges in distinguishing OA symptoms from MS manifestations, underscores the need for further research to elucidate the pathophysiological mechanisms and interactions between these conditions. Additional studies are warranted to enhance clinical management and improve outcomes for individuals with MS and co-existing OA.

## 1. Introduction

Multiple sclerosis (MS) is a chronic, neurodegenerative, autoimmune condition affecting more than 2 million individuals worldwide. It is characterized by progressive neural degeneration and neuroinflammation causing a wide range of sensory, motor, cognitive, and psychological disturbances [1]. Although researchers have delved into the intricate neurological manifestations and underlying biological mechanisms of the disease and have managed to significantly increase life the expectancy of people with MS (PwMS) through targeted pharmacotherapeutic interventions, there are several additional aspects, which, collectively, seriously complicate the quality of life (QoL) in these individuals [1].

Beyond the cognitive and psychosocial burden of the disease, the high number of comorbidities and related conditions in PwMS can negatively impact their overall QoL. Common conditions seen in PwMS, which have been a topic of increasing interest in the research field of MS, include cognitive and cardiovascular comorbities, as well as auto-immune or inflammatory degenerative joint diseases (DJDs) [2,3,4,5]. Arthritis is one such frequently occurring non-autoimmune, whole-joint DJD, affecting approximately 21.2% of the global population [2]. A significant percentage of PwMS experience arthritis, with the most frequently occurring types being rheumatoid arthritis (hazard ratio: 1.77), psoriatic arthritis, and osteoarthritis (OA) [3,4,5].

OA, in particular, is a prevalent synovial condition seen in the MS population, characterized by degeneration and loss of articular cartilage, subchondral bone and synovium disorder, and soft tissue degeneration (e.g., meniscal degeneration, inflammation, and fibrosis of the infrapatellar fat pad [2,6]. In spite of the increased occurrence of OA in PwMS, very limited research exists in the current literature, regarding its specific prevalence in this population and its potential association with demographic and MS-specific characteristics [7]. Research has identified the existence of several possible contributors, which could increase the risk of OA occurrence in PwMS. To begin with, over-use of several joints of the lower body and changes in the gait patterns in PwMS may elevate the risk of cartilage degradation and OA. Moreover, in more severe cases of MS, where individuals rely on manual wheelchair use on a daily basis, the upper body joints may also experience excessive wear and tear [7,8].

Additionally, the latest literature suggests that MS-specific characteristics and common comorbidities (such as disease gender bias, diabetes, and obesity) may serve as risk factors for OA [9,10]. More specifically, gender is a well-known risk factor for both MS and OA, as in both conditions, females are more commonly affected than males [9,10]. More specifically, in both diseases, there is a higher prevalence in the female population. Moreover, there is an increased risk of developing OA in both the MS and general population, attributed to the normal wear and tear of the joints. In the case of MS, in particular, individuals often experience an exacerbation of OA symptoms with aging [11]. Furthermore, PwMS are prone to increased BMI [9] as well as a higher prevalence and risk of diabetes mellitus type 2 [12] compared to the general population, both of which are known to increase the risk of developing OA. Obesity is an important risk factor for OA development, particularly in the knees and hips, since these joints bear the majority of the body’s weight. Increased BMI leads to an elevation in the mechanical load on the joint, which, in turn, prompts cartilage degeneration [10]. In the case of PwMS, restricted mobility may result in increases in BMI, which may lead to a higher risk of OA.

Taking into consideration the high prevalence of auto-immune arthritis in the MS population, the heavy burden of OA in PwMS, and the gap in the current literature regarding the prevalence and the possible association of OA with MS, the aim of the study was two-fold. First, we sought to estimate the prevalence of OA in the MS population, and second, we sought to investigate the relevant risk factors and potential association between OA and demographics and MS-related/specific characteristics. By shedding light on these critical aspects, this study aimed to contribute to a more comprehensive understanding of the interplay between MS and OA, ultimately informing more appropriate intervention approaches for PwMS.

## 2. Methods

### 2.1. Standard Protocol Approvals, Registrations

The pre-specified protocol of the present systematic review and meta-analysis was registered in the Open Search Foundation (OSF) (Registration: osf.io/yad7f). The meta-analysis is reported according to the updated Preferred Reporting Items for Systematic Reviews and Meta-Analyses (PRISMA) guidelines [9,13] and was written according to the Meta-analysis of Observational Studies in Epidemiology (MOOSE) proposal [14]. This study did not require ethical board approval or written informed consent according to the study design (a systematic review and meta-analysis).

### 2.2. Data Sources, Searches and Study Selection

Potentially eligible studies reporting on cases of arthritis or OA in patients with MS were identified via a systematic literature search of the MEDLINE and Scopus databases.

Queries included combinations of the following terms: “multiple sclerosis”, “arthritis”, “osteoarthritis”, “arthrosis”, “osteoarthrosis”, and “degenerative joint disease”. The complete search algorithms used are provided in the Supplementary Materials. No language or other restrictions were applied. The search spanned from the inception of each electronic database to 12 April 2024.

Potentially eligible studies included clinical trials, registries and population-based studies as well as observational cohort studies. Based on the Population, Intervention, Comparison, Outcomes (PICO) search strategy, the search was structured as follows: P: Adults with MS; I: N/A; C: N/A; O: Cases of Arthritis or OA.

Per the study protocol, we excluded studies (1) with participants under 18 years of age; (2) on those with rheumatoid arthritis; (3) on those with psoriasic arthritis; (4) on those with ankylosing spondylitis; (5) on those with reporting outcomes not aligned with our inclusion criteria; (6) case series or reports, commentaries, narrative, systematic or scoping reviews, editorials, non-peer reviewed studies, pre-prints and conference abstracts. In case of overlapping data between studies, the study with the largest dataset was retained. Studies where then assessed by two independent reviewers (VG and VS) and any disagreements were resolved by the corresponding author (SG).

### 2.3. Quality Control, Bias Assessment and Data Extraction

The risk of bias for relevant domains in each included study was assessed using the Risk of Bias in Non-randomized Studies of Interventions (ROBINS-I) tool [14]. Two independent reviewers (VG and VS) conducted quality control and bias assessment with disagreements being settled by the corresponding author (SG). Data from individual studies, including author names, date of publication, study design, country, event type (arthritis, OA), and patient characteristics (demographic and disease specific), were extracted in structured reports for further analyses.

### 2.4. Outcomes

An aggregate data meta-analysis was performed with the inclusion of the identified population-based studies or registries, and observational cohort studies.

The predefined primary outcome measure was the pooled prevalence of OA in people with MS. Secondary outcomes of interest were (a) the relevant risk of OA in the MS population and (b) the potential associations of demographic characteristics and MS-related characteristics with the events of OA in the MS population.

### 2.5. Statistical Analysis

The pooled prevalence and corresponding 95% confidence interval (95% CI) were calculated for each dichotomous outcome using the random effects model of meta-analysis (DerSimonian and Laird) with the implementation of the Freeman–Tukey variance-stabilizing double-arcsine transformation [12,14,15,16]. Heterogeneity was assessed using the I^2^ index, while subgroup differences were assessed using the Q test, with the significance level for all tests set at 0.1 [16]. The significance level for the Q statistic was set at 0.1 [16]. Publication bias was assessed via funnel plot inspection and with Egger’s linear regression test using the corresponding z-test with the significance level set at 0.5 [17,18]. Statistical analysis, figure, and plot production was performed using R.4.1.1 (BigSur) for IOS [R studio/R Meta package].

## 3. Results

### 3.1. Literature Search Outcomes

**Figure 1:**
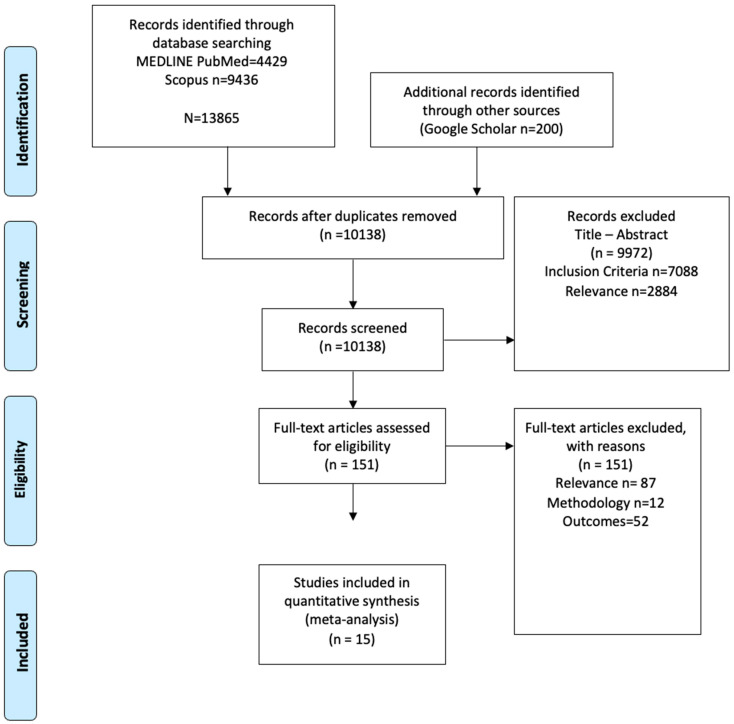
Prisma flowchart.

The systematic literature search yielded 13,685 results. After duplicate removal and the application of the predefined inclusion–exclusion criteria, 151 records were examined in their full-text form. Finally, 15 studies [4,19,20,21,22,23,24,25,26,27,28,29,30,31,32] were deemed eligible for inclusion in synthesis (Figure 1).

### 3.2. Quality Control

**Figure 2:**
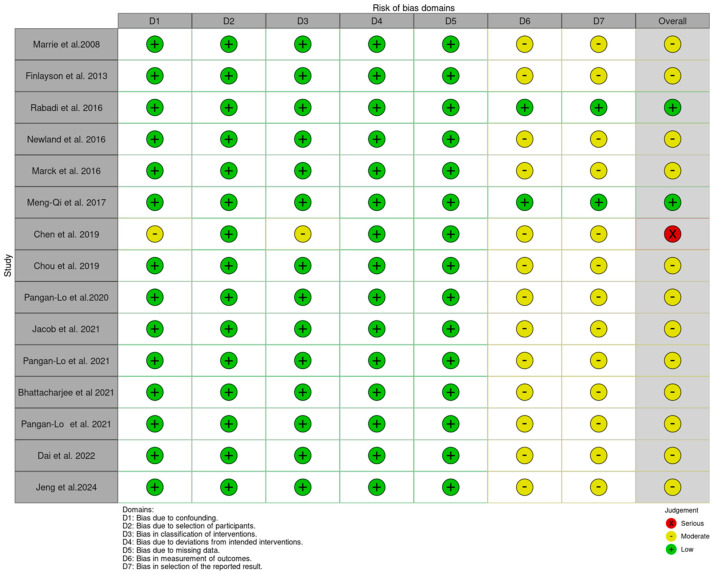
Risk of bias traffic plot [4,20,21,22,23,24,25,26,27,28,29,30,31,32,33].

**Figure 3:**
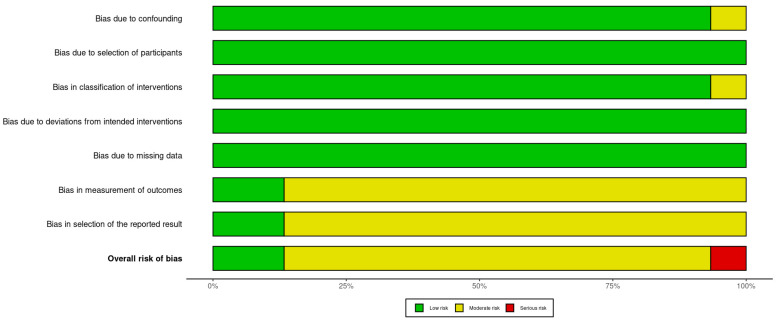
Risk of bias summary plot.

Assessment of the included studies using the ROBINS-I (https://mcguinlu.shinyapps.io/robvis/, accessed on 1 August 2024) indicated a moderate risk of bias, mainly due to the measurement of outcomes and selection of the reported results (Figure 2 and Figure 3).

### 3.3. Primary Outcome

A total of 29,362 people with MS were included in the analysis. In total, 73.09% of the sample was female with a mean age of 51.2 years and a mean disease duration of 15.9 years (Table 1).

**Figure 4:**
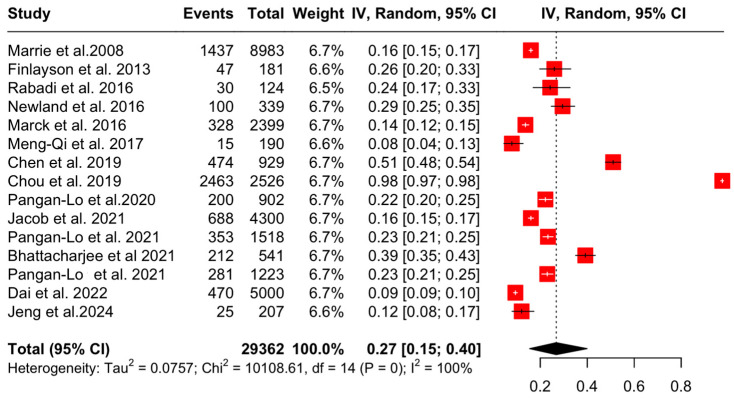
Forest plot: prevalence of OA in PwMS [4,20,21,22,23,24,25,26,27,28,29,30,31,32,33].

The aggregated pooled proportion of OA was 27% (95% CI: [15, 40], I^2^ = 99.9%, *p* = 0.00) (Figure 4).

**Figure 5:**
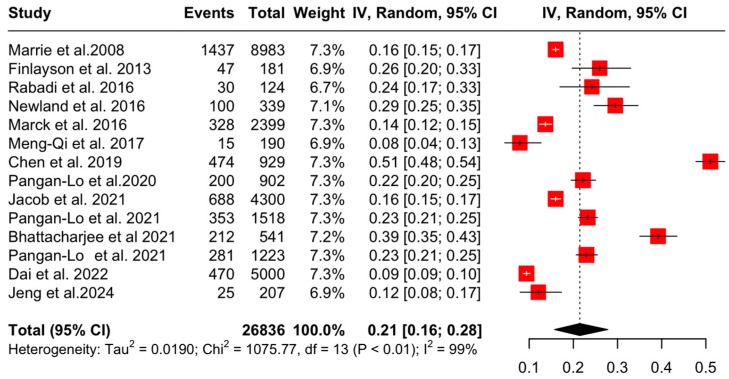
Forest plot: sensitivity analyses of OA prevalence in PwMS [4,20,21,22,23,24,25,27,28,29,30,31,32,33].

Subsequently, sensitivity analyses after the exclusion of one study (Chou et al. 2019 [26], due to a high degree of risk of bias) showed that the aggregated pooled proportion of OA was 21% (95% CI: [16, 28], I^2^ = 98.8%, *p* = 0) (Figure 5).

### 3.4. Secondary Outcomes

**Figure 6:**
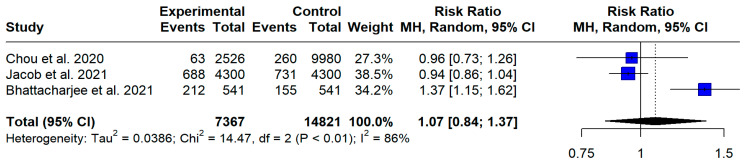
Forest plot: risk ratio of OA in PwMS [26,28,30].

Three studies [25,27] reported events of OA in people with and without MS. The aggregated risk ratio of OA in people with MS versus controls (people without MS) was found to be 1.07 (95% CI [0.84, 1.37], I^2^ = 86.2%, *p* < 0.01) (Figure 6). The fact that the lower confidence interval is less than 1.0 and the aggregated RR is greater than 1.0 indicates that, based on the provided data, the risk of OA does not differ between the MS and control population.

The relationship between the pooled proportion of those with OA and demographic- and disease-specific characteristics was examined using meta-regression techniques.

Neither the participants’ age nor disease duration was found to be associated with the pooled proportion of OA patients in the MS population (β = −0.01, *p* = 0.31 and β = −0.00, *p* = 0.85, respectively).

### 3.5. Publication Bias

Publication bias was assessed via funnel plot inspection (Supplementary Figure S1), as well as via Egger’s linear regression test (β = 8.3, *p* = 0.53). There was a high degree of publication bias as shown by the funnel plot’s asymmetry.

## 4. Discussion

This systematic review and meta-analysis aimed at investigating the prevalence of OA in PwMS as well as the risk factors and potential link between OA development and demographic- and MS-related characteristics. A total of 15 studies and 29,362 PwMS were analyzed in this systematic review. With regard to the primary objective of this study, the aggregated pool prevalence of osteoarthritis in the MS population was found to be 27%. Following sensitivity analyses, to consider the influence of potential bases, the prevalence was estimated to be 21%.

As far as the secondary objectives of this meta-analysis are concerned, this study estimated that the relative risk of OA in PwMS compared to that in controls was 1.07, with the confidence interval being below the 1.0 cut off point, indicating a lack of significant risk of OA in PwMS versus controls. Nonetheless, it is crucial to approach this finding with caution because of the increased heterogeneity and the possibility of residual confounding factors. The absence of a significant difference might be attributed to differences in participants’ demographics, the study design, or the diagnostic criteria for OA in the included studies.

OA entails complex mechanisms characterized by gradual articular cartilage degeneration, remodeling of the underlying bone, and synovitis [33]. Histological examinations of OA joints revealed synovial hypertrophy and hyperplasia with increased lymphocyte and macrophage recruitment, as well as angiogenesis and proliferation of fibroblasts [33,34]. At the same time, angiogenesis and proliferation of fibroblasts are prominent features [34]. Inside the osteochondral unit, there is an increased loss of chondrocytes and the extracellular matrix, coupled with subchondral bone remodeling, which leads to sclerosis and osteophyte formation [34]. These pathological alterations play a part in the joint stiffness and chronic pain linked to OA. For example, in the knee join, the infrapatellar fat pad (IFP), located between the femur, patella, tibia and meniscus, is commonly affected by osteoarthritis (OA), resulting in fibrosis, inflammation and/or hypertrophy [35].

Etiologically, OA can be classified as primary or secondary. If it is unrelated to a prior event or condition, it is considered primary OA, while if it is the result of trauma or a pre-existing disease/condition, it is classified as secondary OA [36]. It can occur in any diarthrodial joint but is most frequently observed in the hip, knee and facet joints of the spines [37]. When considering that, in the general population, OA accounts for 2–3% of all disabilities [37] and that PwMS have higher levels of disability than the general population, a high prevalence of OA in the MS population can have catastrophic effects for the patients. Considering that OA already represents a common cause of disability for a significant portion of the general population, its elevated prevalence in the MS population may aggravate functional restrictions and degrade the overall QoL of the affected individuals.

The foremost limitations imposed by OA are pain and activity withdrawal [38]. Furthermore, from an embiomechanical standpoint, OA results in reduced hip or knee motion, which subsequently elevates pelvic motion, affecting the natural mobility of the lumbar spine and resulting in lumbar pain [7]. In the case of MS with comorbid OA, where PwMS require more time for the completion of a single walking cycle and have a prolonged double-support walking phase, a shorter stride length and lower gait velocity, the interaction between these two disturbances can further limit the person’s mobility [8].

There are several hypotheses regarding the etiology underlying the high prevalence of simultaneous MS and OA: First, MS and OA are both gender-biased diseases. The ratio of female to male patients regarding MS is as high as 4:1 [39], while for OA, the ratio is approximately 2:1 (based on age) [11]. Second, its has been reported that people with Diabetes Mellitus type II have a higher risk of presenting with OA due to an altered glucose mechanism and chondrocyte function disorder due to altered glucose concentration [40,41,42,43]. Taking into account that the prevalence of T2DM in the MS population is approximately 5% [10], there may be a potential association between OA in PwMS with comorbid T2DM.

Additionally, there is some evidence of peripheral blood circulating T and B cell involvement in the pathogenesis of OA. T cells, especially CD4+ T helper cells, have been shown to produce pro-inflammatory cytokines, which contribute to the inflammatory environment in OA. In their recent study, Zhu and colleagues reported a normal proportion of CD8+ T cells but a reduced proportion of naïve CD8+ T cells (CD3+, CD8+, CCR7+, CD45RA+) in patients with OA, indicating that aging may affect the immune response and potentially exacerbate joint degeneration [44]. Additionally, several cytokines such as IL-6 and TNF-alpha cytokines have the potential promote synovitis, resulting in further joint inflammation and cartilage degradation. Data from epidemiological studies suggest that in patients with OA, there are elevated levels of IL-6 and C-reactive protein (CRP), which have been found to be related to the disease’s progression [45]. Apart from T cells, B cells may also be implicated in OA, via the production of antibodies against joint antigens, which can lead to an autoimmune-like responses. B cells can accumulate in the synovial tissue of OA patients, which might, therefore, underline their involvement in the chronic inflammatory state.

In the context of MS, CD8+ T cells are known to be involved in central nervous system damage, but they may also contribute to the pathophysiology of OA [45,46]. Increased amounts of CRP and IL-6, both of which are commonly seen in OA patients, have been associated with disease progression and may reflect ongoing immune activation [44,45]. The presence of high levels of IL-6 in the serum of PwMS, especially those with a longer disease duration, suggests a systemic inflammatory state that could exacerbate joint degeneration [47]. Lastly, the use of glucocorticosteroids, commonly prescribed for managing MS relapses, has been associated with secondary OA and other joint complications, such as osteonecrosis and glucocorticoid-induced avascular bone necrosis [48,49].

### 4.1. Clinical Implications

The findings of this meta-analysis underscore several crucial implications for clinical practice. To begin with, bearing in mind the high occurrence of OA in PwMS, routine screening for OA symptoms should be integrated into the regular clinical assessment of MS patients, especially at the more advanced stages of the disease. Several clinical non-interventional assessment methods have been proposed for the evaluation of the risk of OA and the degree of OA in non-neurological patients. The timed up and go test, is a quick and relatively easy test assessing functional mobility, has been identified as a functional manifestation of early knee OA and can be combined with neuropsychological assessments (e.g., the timed up and go cognitive test where the patient has to perform the normal assessment while reciting the days of the week in reverse or subtracting 7 from 100) [50,51]. Moreover, the five-repetition sit-to-stand test has good test–retest reliability when used in neurological patients [52] and has been correlated with knee muscle strength, pain, stiffness and physical function in older adults with knee OA [53]. It is worth noting that even though these tests have not been validated in the MS population, their implementation poses low to no risk of harm to PwMS and can provide useful information to clinicians not only regarding the risk and degree of OA, but also for their overall functional mobility [54].

The rationale behind this recommendation lies in the fact that early identification and management of OA can prevent additional damage and lead to better clinical outcomes. Management strategies could entail tailored physical therapy programs that target both MS and OA symptoms, weight management strategies to minimize joint load, as well as pharmacological interventions to address pain and inflammation. This highlights the need for a multidisciplinary approach to patient care, since addressing the functional limitations and pain associated with OA in PwMS may consequently improve the overall QoL of these patients.

Moreover, healthcare providers should be conscious of the possible effects of long-term MS medication use on joint health. Medications such as corticosteroids, often prescribed for management of MS relapses, may be linked to bone density loss and elevate the risk of OA. Monitoring bone density and exploring different intervention strategies, when necessary, could reduce these risks.

Finally, the results suggest the need for more research to explore the underlying mechanisms linking MS and OA. Longitudinal studies with larger sample sizes and more rigorous control for confounding variables are vital to better comprehending the causal relationships and identify the underlying pathological and mechanical pathways through which MS may contribute to OA development. By elucidating these connections, researchers can develop targeted interventions to prevent or delay the onset or reduce the burden of OA in individuals with MS.

### 4.2. Strengths and Limitations

To our knowledge, this is the first attempt to systematically review and analyze the prevalence of OA in the MS population. However, the insights provided by this study should be interpreted in conjunction with the following limitations, in order to warrant consideration of contextualizing the findings and using the results to guide future research endeavors. To begin with, there is a high degree of heterogeneity between the included studies. Second, the majority of the studies did not report key data such as EDSS and affected OA joint data. Third, there is evidence regarding the potential contribution of glucocorticosteroid treatment for MS to OA-related symptomatology, but additional research is needed to distinguish the specific effects of MS medications on OA risk, considering potential confounding factors such as disease severity and treatment duration. Finally, there was a high degree of publication bias across the included studies. Nevertheless, while the existing literature has attempted to explore the association between MS and OA, further studies are needed to elucidate the pathophysiological mechanisms linking these conditions for optimal patient care and management strategies for MS and concurrent OA.

## 5. Conclusions

OA is one of the most common disabling joint degenerative conditions. The results of this study found that PwMS have a 21–27% prevalence of OA. Given the implications pertaining to the pain and mobility domains, as well as the masking of OA symptoms from MS physicians, further studies are needed to better understand the pathophysiological mechanisms and interactions between the two conditions. By enriching our knowledge on the association between these two variables, we can ultimately improve the QoL of PwMS and provide more targeted and efficient intervention strategies.

## Figures and Tables

**Table 1:** Study characteristics.

Author	Region	Study Type	Sample	OA Cases	Mean Age	% of Female Patients	EDSS	Disease Duration	% of RRMS Patients
Marrie et al. 2008 [4]	USA	Observational	8983	1437	47.4	75.8		16.2	88.5
Finlayson et al. 2013 [20]	Canada	Observational	181	47	55.5	79.1		14.6	53.4
Rabadi et al. 2016 [21]	USA	Observational	124	30	49	26	4.6	24	49
Newland et al. 2016 [22]	USA	Cross-Sectional	339	100	48.2	89		9.9	65
Marck et al. 2016 [23]	Australia	Observational	2399	328	45.5	82.3		6.1	
Meng-Qi et al. 2017 [24]	China	Observational	190	15	54.9	67.8	5.8	19.3	22
Chen et al. 2019 [25]	Australia	Observational	929	474	51.6	80.6		13	
Chou et al. 2019 [26]	UK	Registry	2526	2463	45.03	70.8			
Pangan-Lo et al. 2020 [27]	Australia	Observational	902	200	55.8	78.6		15.4	87.4
Jacob et al. 2021 [28]	Germany	Cohort	4300	688	43.6	69.3			
Pangan-Lo et al. 2021 [29]	Australia	Registry	1518	353	55.7	79.6		20.5	
Bhattacharjee et al. 2021 [30]	USA	Observational	541	212		74.3			
Pangan-Lo et al. 2021 [31]	Australia	Observational	1223	281	56.1	78.7		15.2	
Dai et al. 2022 [32]	USA	Cohort	5000	470	52.9	75.2			
Jeng et al. 2024 [33]	USA	Observational	207	25	49.6	74.3		12.9	93.2

Note. EDSS: expanded disability severity scale; RRMS: relapsing remitting multiple sclerosis.

## Data Availability

All data generated or analyzed during this study are included in this article and its Supplementary Information files.

## Supplementary Materials

The following supporting information can be downloaded at https://www.mdpi.com/article/10.3390/jcm13175015/s1: Search algorithm; Supplementary Figure S1: Funnel plot for publication bias of reported arthritis proportion in MS population.

## References

[1] Wallin M.T., Culpepper W.J., Nichols E., Bhutta Z.A., Gebrehiwot T.T., Hay S.I., Khalil I.A., Krohn K.J., Liang X., Naghavi M. (2019). Global, regional, and national burden of multiple sclerosis 1990–2016: A systematic analysis for the global burden of disease study 2016. Lancet Neurol..

[2] Centers for Disease Control and Prevention, National Center for Chronic Disease Prevention and Health Promotion, Division of Population Health. https://www.cdc.gov/nccdphp/index.html.

[3] Safiri S., Kolahi A.-A., Smith E., Hill C., Bettampadi D., Mansournia M.A., Hoy D., Ashrafi-Asgarabad A., Sepidarkish M., Almasi-Hashiani A. (2020). Global, regional and national burden of osteoarthritis 1990-2017: A systematic analysis of the Global Burden of Disease Study 2017. Ann. Rheum. Dis..

[4] Marrie R., Horwitz R., Cutter G., Tyry T., Campagnolo D., Vollmer T. (2008). Comorbidity, socioeconomic status and multiple sclerosis. Mult. Scler..

[5] Tseng C.-C., Chang S.-J., Tsai W.-C., Ou T.-T., Wu C.-C., Sung W.-Y., Hsieh M.-C., Yen J.-H. (2016). Increased incidence of rheumatoid arthritis in multiple sclerosis: A nationwide cohort study. Medicine.

[6] Johnson V.L., Hunter D.J. (2014). The epidemiology of osteoarthritis. Best Pract. Res. Clin. Rheumatol..

[7] Bejek Z., Paróczai R., Illyés Á., Kiss R.M. (2006). The influence of walking speed on gait parameters in healthy people and in patients with osteoarthritis. Knee Surg. Sport. Traumatol. Arthrosc..

[8] Dujmovic I., Radovanovic S., Martinovic V., Dackovic J., Maric G., Mesaros S., Pekmezovic T., Kostic V., Drulovic J. (2017). Gait pattern in patients with different multiple sclerosis phenotypes. Mult. Scler. Relat. Disord..

[9] Giannopapas V., Stefanou M.-I., Smyrni V., Kitsos D.K., Kosmidou M., Stasi S., Chasiotis A.K., Stavrogianni K., Papagiannopoulou G., Tzartos J.S. (2024). Waist Circumference and Body Mass Index as Predictors of Disability Progression in Multiple Sclerosis: A Systematic Review and Meta-Analysis. J. Clin. Med..

[10] Nedunchezhiyan U., Varughese I., Sun A.R., Wu X., Crawford R., Prasadam I. (2022). Obesity, Inflammation, and Immune System in Osteoarthritis. Front. Immunol..

[11] Tschon M., Contartese D., Pagani S., Borsari V., Fini M. (2021). Gender and Sex Are Key Determinants in Osteoarthritis Not Only Confounding Variables. A Systematic Review of Clinical Data. J. Clin. Med..

[12] Giannopapas V., Palaiodimou L., Kitsos D., Papagiannopoulou G., Stavrogianni K., Chasiotis A., Kosmidou M., Tzartos J.S., Paraskevas G.P., Bakalidou D. (2023). The Prevalence of Diabetes Mellitus Type II (DMII) in the Multiple Sclerosis Population: A Systematic Review and Meta-Analysis. J. Clin. Med..

[13] Page M.J., McKenzie J.E., Bossuyt P.M., Boutron I., Hoffmann T.C., Mulrow C.D., Shamseer L., Tetzlaff J.M., Akl E.A., Brennan S.E. (2021). The PRISMA 2020 statement: An updated guideline for reporting systematic reviews. BMJ.

[14] Sterne J.A.C., Hernán M.A., Reeves B.C., Savović J., Berkman N.D., Viswanathan M., Henry D., Altman D.G., Ansari M.T., Boutron I. (2016). ROBINS-I: A tool for assessing risk of bias in non-randomised studies of interventions. BMJ.

[15] Stefanou M.I., Palaiodimou L., Katsanos A.H., Milionis H., Kosmidou M., Lambadiari V., Halvatsiotis P., Ferentinos P., Andreadou E., Marinos G. (2022). The effects of HMG-CoA reductase inhibitors on disease activity in multiple sclerosis: A systematic review and meta-analysis. Mult. Scler. Relat. Disord..

[16] Freeman M.F., Tukey J.W. (1950). Transformations related to the angular and the square root. Ann. Math. Stat..

[17] Tsivgoulis G., Katsanos A.H., Köhrmann M., Caso V., Perren F., Palaiodimou L., Deftereos S., Giannopoulos S., Ellul J., Krogias C. (2019). Duration of Implantable Cardiac Monitoring and Detection of Atrial Fibrillation in Ischemic Stroke Patients: A Systematic Review and Meta-Analysis. J. Stroke.

[18] Borenstein M., Higgins J.P. (2013). Meta-analysis and subgroups. Prev. Sci..

[19] Egger M., Smith G.D., Schneider M., Minder C. (1997). Bias in meta-analysis detected by a simple, graphical test. BMJ.

[20] Finlayson M., Preissner K., Cho C. (2013). Impact of comorbidity on fatigue management intervention outcomes among people with multiple sclerosis: An exploratory investigation. Int. J. MS Care.

[21] Rabadi M.H., Aston C.E. (2016). Effect of Chronic Medical Conditions in Veterans with Multiple Sclerosis on Long-Term Disability. Med. Sci. Monit..

[22] Newland P.K., Lorenz R., Budhathoki C., Jensen M.P. (2016). The Presence of Symptoms With Comorbid Conditions in Individuals With Multiple Sclerosis (MS). Clin. Nurs. Res..

[23] Marck C.H., Neate S.L., Taylor K.L., Weiland T.J., Jelinek G.A. (2016). Prevalence of Comorbidities, Overweight and Obesity in an International Sample of People with Multiple Sclerosis and Associations with Modifiable Lifestyle Factors. PLoS ONE.

[24] Na M.Q., Deng S., Yan X.L., Wang Y.L. (2017). Risk factors analysis for low back pain in patients with multiple sclerosis. China J. Orthop. Traumatol..

[25] Chen J., Taylor B., Winzenberg T., Palmer A.J., Kirk-Brown A., van Dijk P., Simpson S.J., Blizzard L., van der Mei I. (2020). Comorbidities are prevalent and detrimental for employment outcomes in people of working age with multiple sclerosis. Mult. Scler. J..

[26] Chou I.J., Kuo C.F., Tanasescu R., Tench C.R., Tiley C.G., Constantinescu C.S., Whitehouse W.P. (2020). Comorbidity in multiple sclerosis: Its temporal relationships with disease onset and dose effect on mortality. Eur. J. Neurol..

[27] Lo L.M.P., Taylor B.V., Winzenberg T., Palmer A.J., Blizzard L., van der Mei I. (2021). Comorbidities contribute substantially to the severity of common multiple sclerosis symptoms. J. Neurol..

[28] Jacob L., Smith L., Koyanagi A., Haro J.M., Konrad M., Tanislav C., Kostev K. (2021). Is there an association between multiple sclerosis and osteoarthritis in Germany? A retrospective cohort study of 8600 patients from Germany. Mult. Scler. J. Exp. Transl. Clin..

[29] Lo L.M.P., Taylor B.V., Winzenberg T., Palmer A.J., Blizzard L., van der Mei I. (2021). Change and onset-type differences in the prevalence of comorbidities in people with multiple sclerosis. J. Neurol..

[30] Bhattacharjee S., Yegezu Z., Kollecas K., Duhrkopf K., Hashemi L., Greene N. (2021). Influence of Comorbidities on Healthcare Expenditures and Perceived Physical and Mental Health Status Among Adults with Multiple Sclerosis: A Propensity Score-Matched US National-Level Study. Clin. Outcomes Res..

[31] Lo L.M.P., Taylor B.V., Winzenberg T., Palmer A.J., Blizzard L., Ahmad H., Hussain M.A., van der Mei I. (2021). Estimating the relative contribution of comorbidities in predicting health-related quality of life of people with multiple sclerosis. J. Neurol..

[32] Dai D., Sharma A., Phillips A.L., Lobo C. (2022). Patterns of Comorbidity and Multimorbidity Among Patients With Multiple Sclerosis in a Large US Commercially Insured and Medicare Advantage Population. J. Health Econ. Outcomes Res..

[33] Jeng B., Huynh T.L., Motl R.W. (2024). Comorbid Conditions and Physical Function in Adults with Multiple Sclerosis. Arch. Phys. Med. Rehabil..

[34] Glyn-Jones S., Palmer A.J., Agricola R., Price A.J., Vincent T.L., Weinans H., Carr A.J.R. (2015). Osteoarthritis. Lancet.

[35] Uhalte E.C., Wilkinson J.M., Southam L., Zeggini E. (2017). Pathways to understanding the genomic aetiology of osteoarthritis. Hum. Mol. Genet..

[36] Buchanan W.W., Kean W.F. (2002). Osteoarthritis IV: Clinical therapeutic trials and treatment. Inflammopharmacology.

[37] Michael J.W.P., Schlüter-Brust K.U., Eysel P. (2010). The epidemiology, etiology, diagnosis, and treatment of osteoarthritis of the knee. Dtsch. Arztebl. Int..

[38] Buckwalter J.A., Martin J.A. (2006). Osteoarthritis. Adv. Drug Deliv. Rev..

[39] Walton C., King R., Rechtman L., Kaye W., Leray E., Marrie R.A., Robertson N., La Rocca N., Uitdehaag B., Van Der Mei I. (2020). Rising prevalence of multiple sclerosis worldwide: Insights from the Atlas of MS, third edition. Mult. Scler..

[40] Schett G., Kleyer A., Perricone C., Sahinbegovic E., Iagnocco A., Zwerina J., Lorenzini R., Aschenbrenner F., Berenbaum F., D’Agostino M.A. (2013). Diabetes is an independent predictor for severe osteoarthritis: Results from a longitudinal cohort study. Diabetes Care.

[41] Yan W., Li X. (2013). Impact of diabetes and its treatments on skeletal diseases. Front. Med..

[42] Velasquez M.T., Katz J.D. (2010). Osteoarthritis: Another component of metabolic syndrome?. Metab. Syndr. Relat. Disord..

[43] Fontanella C.G., Belluzzi E., Pozzuoli A., Favero M., Ruggieri P., Macchi V., Carniel E.L. (2022). Mechanical behavior of infrapatellar fat pad of patients affected by osteoarthritis. J. Biomech..

[44] Rosa S.C., Goncalves J., Judas F., Mobasheri A., Lopes C., Mendes A.F. (2009). Impaired glucose transporter-1 degradation and increased glucose transport and oxidative stress in response to high glucose in chondrocytes from osteoarthritic versus normal human cartilage. Arthritis Res. Ther..

[45] Zhu W., Zhang X., Jiang Y., Liu X., Huang L., Wei Q., Huang Y., Wu W., Gu J. (2020). Alterations in peripheral T cell and B cell subsets in patients with osteoarthritis. Clin. Rheumatol..

[46] Greene M., Loeser R. (2015). Aging-related inflammation in osteoarthritis. Osteoarthr. Cartil..

[47] Salou M., Nicol B., Garcia A., Laplaud D.A. (2015). Involvement of CD8(+) T Cells in Multiple Sclerosis. Front. Immunol..

[48] Stelmasiak Z., Kozioł-Montewka M., Dobosz B., Rejdak K., Bartosik-Psujek H., Mitosek-Szewczyk K., Belniak-Legieć E. (2000). Interleukin-6 concentration in serum and cerebrospinal fluid in multiple sclerosis patients. Med. Sci. Monit..

[49] Motta F., Timilsina S., Gershwin M.E., Selmi C. (2022). Steroid-induced osteonecrosis. J. Transl. Autoimmun..

[50] Chan K.L., Mok C.C. (2012). Glucocorticoid-induced avascular bone necrosis: Diagnosis and management. Open Orthop. J..

[51] Shimizu H., Shimoura K., Iijima H., Suzuki Y., Aoyama T. (2022). Functional manifestations of early knee osteoarthritis: A systematic review and meta-analysis. Clin. Rheumatol..

[52] Çekok K., Kahraman T., Duran G., Çolakoğlu B.D., Yener G., Yerlikaya D., Genç A. (2020). Timed Up and Go Test With a Cognitive Task: Correlations With Neuropsychological Measures in People With Parkinson’s Disease. Cureus.

[53] Silva P.F., Quintino L.F., Franco J., Faria C.D. (2014). Measurement properties and feasibility of clinical tests to assess sit-to-stand/stand-to-sit tasks in subjects with neurological disease: A systematic review. Braz. J. Phys. Ther..

[54] Khuna L., Soison T., Plukwongchuen T., Tangadulrat N. (2024). Reliability and concurrent validity of 30-s and 5-time sit-to-stand tests in older adults with knee osteoarthritis. Clin. Rheumatol..

