# Supplementary material for: Osteoarthritis in People with Multiple Sclerosis: A Systematic Review and Meta-Analysis
Source: J Clin Med. 2024 Aug 24;13(17):5015. doi: 10.3390/jcm13175015 (PMC11396250; doi:10.3390/jcm13175015)

Supplementary Materials

Search Algorithm

MEDLINE PubMed search algorithm

(multiple sclerosis) AND ((osteoarthritis) OR (osteoarthrosis) OR (arthritis) OR (degenerative joint disease))

Scopus search algorithm

("multiple sclerosis" AND "osteoarthritis") OR ("multiple sclerosis" AND "osteoarthrosis") OR ("multiple sclerosis" AND "arthritis") OR ("multiple sclerosis" AND "degenerative joint disease")

Supplementary Figures Legends

Supplementary Figure S1: Funnel plot for publication bias of reported arthritis proportion in the MS population

Supplementary Figure S1

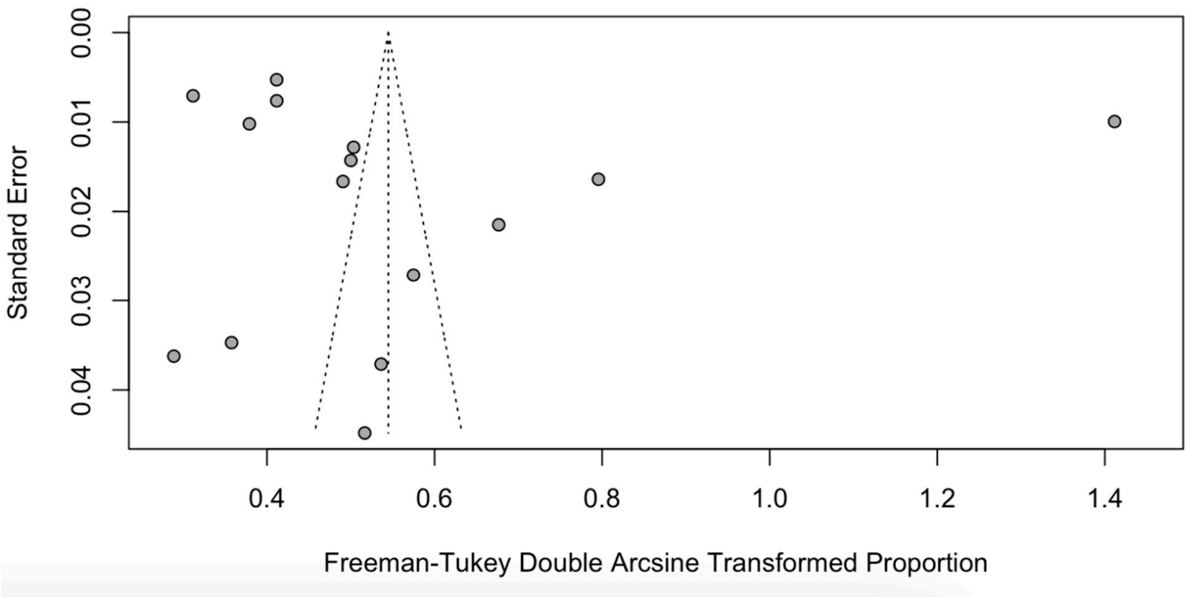

Supplement: Supplementary file 1 [file jcm-13-05015-s001.zip › jcm-3159952-supplementary.pdf]
